# Diversely positive-charged gold nanoparticles based biosensor: A label-free and sensitive tool for foodborne pathogen detection

**DOI:** 10.1016/j.fochx.2019.100052

**Published:** 2019-08-23

**Authors:** Tong Bu, Pei Jia, Jinghan Liu, Yingnan Liu, Xinyu Sun, Meng Zhang, Yongming Tian, Daohong Zhang, Jianlong Wang, Li Wang

**Affiliations:** College of Food Science and Engineering, Northwest A&F University, Yangling 712100, Shaanxi, China

**Keywords:** Label-free, Positively charged AuNPs, Lateral flow strip, Sensitive detection, *Salmonella enteritidis*, *Escherichia coli* O157

## Abstract

Gold nanoparticles (AuNPs)-based lateral flow strip (LFS) enables a quick screening of foodborne bacteria in point-of-care (POC) diagnostics but suffers from the cumbersome labeling of antibodies and poor sensitive performance. Here, we innovated a label-free immunoassay to detect pathogenic bacteria by introducing diversely positive charges functionalized AuNPs ((+) AuNPs). The (+) AuNPs can be loaded on negatively charged bacteria via electrostatic interaction, leading to a color labeling to target bacteria. Afterward, the (+) AuNPs-bacteria complex can be specifically captured by monoclonal antibody (McAb) immobilized on test (T)-line. Under optimum conditions, the proposed LFS exhibited a lowest detectable concentration of 10^3^ CFU/mL for *Salmonella enteritidis* (*S. enteritidis*) and could be well applied in drinking water, lettuce and pork samples. Moreover, this novel (+) AuNPs-LFS possessed a universal applicability, which could also be used for detecting *Escherichia coli* O157 (*E. coli* O157) with a superior sensitivity (10^4^ CFU/mL) and specificity.

## Highlights

• A label-free, non-paired antibodies dependent biosensor was constructed.
• (+) AuNPs were employed to generate signal and capture bacteria in the new LFS.
• This work possesses a desirable universality for other pathogens.

## 1 Introduction

As one of the most common commercial point-of-care (POC) devices, lateral flow strip (LFS) using gold nanoparticles (AuNPs) labeled antibodies has been extensively applied into the pathogen monitoring field (Cheng et al., 2017, Cho et al., 2015, Kong et al., 2017), benefiting from the effective cost, small amount of the sample and handy implementation (Niu et al., 2014, Zhang et al., 2015). Still, current AuNPs-based LFS is limited to the detection of bacteria with relatively high concentrations in samples. Thus, the poor detection sensitivity is a major restriction of this traditional sensor (Nash, Waitumbi, Hoffman, Yager, & Stayton, 2012). In order to improve the performance of AuNPs-based LFS biosensor for the detection of microorganisms, two problems need to be solved: (1) how to simply construct a recognition carrier capable of fast and robust interaction with bacteria and also exhibit high sensitivity; (2) endurance of antibody labeled probes to rough conditions (such as organic extracts) is the other key issue to consider.

Commonly, the detection limits of AuNPs-based LFS are primarily determined by the binding capacity of Au labeled antibodies for the target analytes (Zhang et al., 2015). Indeed, label-free AuNPs inherently can be used as probes for interaction with biological molecules, non-specifically, leading to a color change (Pan et al., 2012). It has already been reported that nanoparticles with different surface properties (e.g. size, shape, surface charge, and coating material) could interact with target substances correspondingly via Van der Waals forces, covalent bond forces, etc (Berry et al., 2005, Li et al., 2015, Wang et al., 2016). Among them, (+) AuNPs is the remarkable one. For example, Miao and her co-workers (Miao et al., 2017) proposed a label-free fluorescence using (+) AuNPs to quench the Ag nanoclusters for detection of microRNA-155. Su and her co-workers (Jiao, Wenjiao, Yun, Ruo, & Yaqin, 2013) developed a direct visualization sensor for detection of lysozyme, in which negatively charged aptamers were adsorbed by (+) AuNPs as a novel signal probe. These approaches avoided the lengthy receptor–traditional negatively charged gold nanoparticles labeling process and could be more stable in complex systems. On the basis of the above mentioned thoughts, considering the fact that most of bacteria have negatively charged surface, the design of a sensitive and label-free LFS based on electrostatic adsorption principle for detecting bacteria is possible. Consequently, unlabeled (+) AuNPs, as powerful sensing elements, have potential to monitor diverse bacteria in LFS biosensor because they can be assembled onto the surface of bacteria and possess the dual functions of receptors and indicators simultaneously.

Herein, the aim of the study was to explore a colorimetric LFS for rapid and sensitive detection of foodborne pathogens in drinking water, lettuce and pork samples using two (+) AuNPs as carriers, i.e. AuNPs coated cysteamine (AuNPs@Cys) and cetyltrimethylammonium bromide (AuNPs@CTAB). Cysteamine was modified on the surfaces of the AuNPs by strong Au–S bonds and the –NH3^+^ terminus of Cys confers positive charges to the AuNPs (Jiao et al., 2013). The positively charged CTAB surfactant acts as a stabilizer to prevent surface oxidation of the particles and to control the overall morphology of the particles (Al-Thabaiti, Obaid, Khan, Bashir, & Hussain, 2015). The (+) AuNPs can combine with negatively charged bacteria to form a complex, which is selectively recognized by the McAb immobilized on *T*-line. The micromorphologies, particle sizes and zeta potentials of the (+) AuNPs were characterized and the detection conditions for target bacteria were optimized. The (+) AuNPs-based strip just using a label-free tracer and one capture antibody can successfully detect target with excellent sensitivity and selectivity, where intensely colored *T*-line can be observed even at a low analyte concentration. In addition, the traditional AuNPs-McAb based LFS was also conducted to compare the performance with the developed biosensor. The extraordinary applicability of versatile positive-charged nanoparticles in LFS biosensor reveals that the (+) AuNPs holds great promise as a universal probe sensing for various bacteria in food safety and early clinical diagnosis.

## 2 Materials and methods

### 2.1 Reagents and chemicals

High specific McAb against *S. enteritidis* flagellin was prepared in our laboratory (Bu et al., 2019). Anti-*E. coli* O157 McAb was obtained from Abcam (Shanghai) Trading Co., Ltd. After characterization through enzyme linked immunosorbent assay (ELISA), the sensitivity of McAb for *S. enteritidis* and *E. coli* O157 detection was 10^3^ CFU/mL and 10^6^ CFU/mL, respectively (Fig. S1). In addition, the anti-*S. enteritidis* McAb showed no cross reactions with other interfering microorganisms (Fig. S2). The Anti-*E. coli* O157 McAb also indicated no cross reactions with other interfering bacteria (Fig. S3). Drinking water (Hangzhou Wahaha Group Co., Ltd.), lettuce and pork samples were obtained from the supermarket (Xi’an, China). For making test strips, nitrocellulose (NC) membranes and glass fibers were all purchased from Shanghai Jinbiao Biological Technology Co., Ltd. Chloroauric acid, sodium citrate, cysteamine and cetyltrimethyl ammonium bromide were supplied from Chengdu XiYa Chemical Technology Co., Ltd. NaBH_4_ and ascorbic acid were provided from Shanghai Aladdin Chemisty Co., Ltd. Luria-Bertani (LB) broth was provided from Qingdao Rishui Biotechnology Co., Ltd. The high speed refrigerated centrifuge (CT14RDⅡ, average radius of rotor (8.3 cm)) was obtained from Xi’an Savis Biotechnology Co., Ltd. (Xi’an, China).

### 2.2 Preparation of the bacterial cultures

In this study, all the bacterial strains were listed in Table S1. They were received from Xi'an Boxu Experimental Technology Co., Ltd. Cultivation of all strains was conducted in LB medium at for 37 °C for 12 h. Finally, the bacteria were diluted with the sterile phosphate buffered solution (PBS) to approximately 10^8^ CFU/mL for further use (Huang, Zhang, Wang, Lai, & Lin, 2018).

### 2.3 Synthesis of AuNPs@Cys probes

The cysteamine-modified AuNPs were synthesized by the previous literature (Li et al., 2017). First, 500 µL of Cys (213 mM) aqueous solution was added quickly to pre-cooled HAuCl_4_ (50 mL, 1.42 mM) with thoroughly stirring for 30 min. Then 12.5 µL of ice-cold NaBH_4_ (10 mM) was added dropwise to the resulting mixture with rapid stirring in the dark for 10 min. After standing for 90 min at room temperature, the mixed solution turned into dark wine, indicating that Cys-capped gold nanoparticles were successfully generated.

### 2.4 Synthesis of AuNPs@CTAB probes

The CTAB-capped gold nanoparticles were fabricated by a classical seed-mediated growth synthesis process (Jana, Gearheart, & Murphy, 2001).

(1) Synthesis of seeds: the pre-cooled HAuCl_4_ (0.5 mM) and trisodium citrate (0.5 mM) with the same volume (10 mL) were mixed and rapidly stirred for 10 min, followed by slow dropwise addition of 0.1 M NaBH_4_ (0.6 mL, ice cold) with continuously stirring for 2 h. Finally, this solution was used as seeds solution for further use;
(2) Preparation of growth solution: 3 g CTAB solid powder was dissolved into 0.5 mM HAuCl_4_ (200 mL) aqueous solution under magnetic stirring. Then the mixture was heated to 50 °C and stirred thoroughly until a stable transparent solution was obtained;
(3) Growth of seeds: 0.1 mL of ascorbic acid (0.1 M) was combined in growth solution (18 mL) with sufficient stirring, followed by addition of 2 mL Au seeds and reacting for 15 min. After incubating for 30 min, the CTAB-capped gold nanoparticles were prepared. Before using, the AuNPs@CTAB colloidal solution should be purified through two sequential centrifugation cycles at 9279 × *g* for 30 min.

### 2.5 LFS membrane preparation

**Scheme 1:**
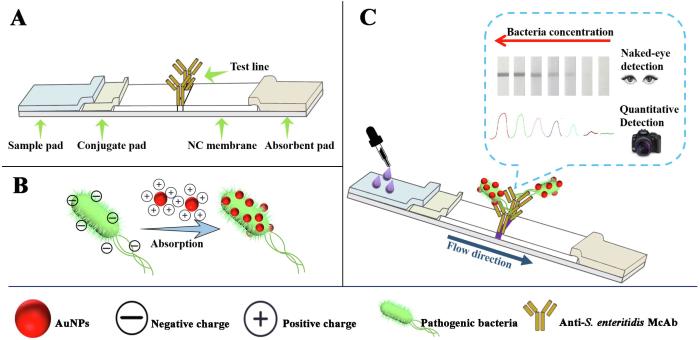
Schematic illustration of (A) the structure of the test strip, (B) combining AuNPs with *S. enteritidis*, (C) AuNPs and *S. enteritidis* flow from conjugate pad to absorbent pad, the appearance of the color of the *T*-line, and the principle of quantitative detection strategy of *S. enteritidis* using camera as readout.

The LFS biosensor had five components: sample pad, conjugate release pad, NC membrane, absorbent pad, and poly (vinyl chloride) (PVC) plate (Scheme 1A). The absorbent pad was used as receptor without further modification. The sample and conjugate pads were blocked by immersing in immunobuffer (that is, PBS + 2% BSA) and then dried overnight at 37 °C. The detection McAb (1.5 mg/mL) was applied onto NC membrane in the amount of 1 μL/cm as the *T*-line, followed by drying at 37 °C for 30 min and then stored under dry condition at 4 °C until be used. After that, all the components were pasted on a PVC plate, overlapping in sequence. Finally, the lateral flow strips were cut into 3 mm width and stored at 4 °C.

### 2.6 Detection of *S*. *enteritidis* and *E. coli* O157

First, AuNPs@Cys (Optical density at 522 nm was 1.10) (40 μL) or AuNPs@CTAB (Optical density at 530 nm was 0.606) (60 μL) was separately incubated with *S. enteritidis* solution (0.01 M PBS) of varying concentrations between 0–10^8^ CFU/mL. Immediately after mixing (10 s), the mixture was deposited onto the sample pad and kept still for 10 min (AuNPs@Cys-LFS) or 15 min (AuNPs@CTAB-LFS). The images of test strips were photographed by a Cannon digital camera and then processed using Image J to analyze the signal intensity of the *T*-line. *E. coli* O157, as another model, was also treated with (+) AuNPs and evaluated using the same procedures as aforementioned.

To confirm the selectivity of the biosensor for *S. enteritidis*, cross reaction experiments were tested at the same bacteria concentration (10^8^ CFU/mL) with four *Salmonella* strains including *S. london*, *S. typhimurium*, *S. paratyphi B* and *S. hadar*, and 5 non-*Salmonella* strains of *S. aureus*, *E. coli* O157, *C. albicans*, *L. monocytogenes* and *C. coli* as the interference bacteria, respectively, using PBS as the negative control. For *E. coli* O157, the same procedures were employed with non-target bacteria (*L. monocytogenes, S. enteritidis*, *S. aureus*, *C. albicans*, *S. hadar* and *C. coli*) that were supplied in the control groups, using PBS as the negative control.

### 2.7 Detection of *S. enteritidis* and *E. coli* O157 in water, lettuce and pork samples

To determine whether the developed device can be applied for bacteria detection from contaminated real samples, drinking water, lettuce and pork were added in different amounts of *S. enteritidis* and *E. coli* O157. Drinking water, lettuce and pork samples were purchased from the supermarket in Xi’an. Firstly, 5.0 g crushed lettuce and pork samples were immersed in 10 mL of PBS by shaking for 30 min, then incubation 4 °C for 12 h. After centrifugation at 6000 rpm for 10 min, the supernatant was collected. Then the drinking water, pretreated lettuce and pork were sterilized by high-temperature sterilization and filtrated (through 0.22 µm sterile filters), each sample was confirmed by plate counting to test the noncontamination of any bacteria (Shukla, Leem, Lee, & Kim, 2014), followed by spiking with different concentrations of *S. enteritidis* and *E. coli* O157 (ranging from 0 to 10^8^ CFU/mL), respectively (Bayrac et al., 2017, Choi et al., 2018). Other steps were identical to the experimental procedures described in Section 2.6.

## 3 Results and discussion

### 3.1 Adsorption mechanism of the (+) AuNPs-based LFS biosensor

As is known to all, most pathogen bacteria have negatively charged surfaces and can be suitable for the interaction with charged AuNPs (Berry et al., 2005). In this work, AuNPs were endowed with positive charge by coating with AuNPs@Cys and AuNPs@CTAB. During the incubation of *S. enteritidis* and AuNPs, *S. enteritidis* can be captured by (+) AuNPs through the electrostatic interaction between positive charged AuNPs and negative charged bacterial surface (Scheme 1B). Driven by the capillary action, the sample pad was soaked in the formed (+) AuNPs-bacteria complex, and migrated along the sample pad to conjugate pad. Afterward, the complex would be retained by the pre-coated detection McAb on NC membrane owing to the specific antibody-antigen interaction between McAb and *S*. *enteritidis*. With the accumulation of complex, the color on *T*-line would be gradually deepened due to the intrinsic absorbance of AuNPs. In this way, for a positive sample, a characteristic color band could be observed. On the other hand, for a negative sample, no band would appear on the *T*-line (Scheme 1C).

### 3.2 Characterizations of AuNPs

**Fig. 1:**
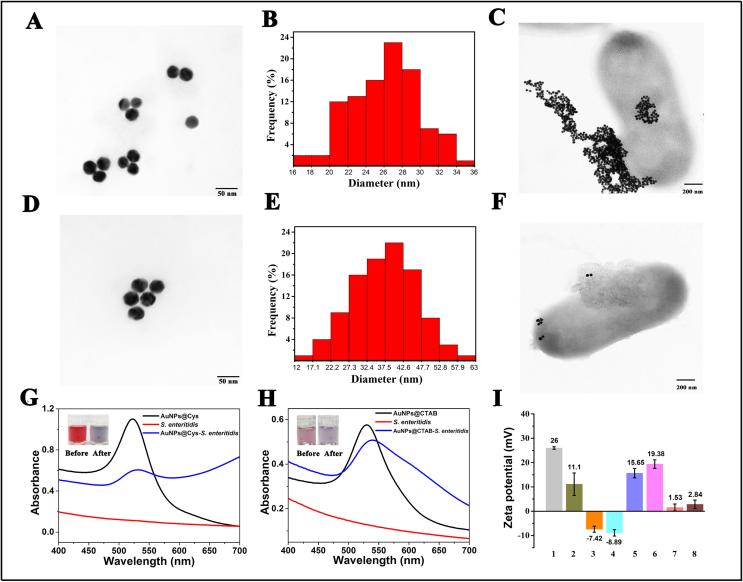
TEM images of (A) AuNPs@Cys and (D) AuNPs@CTAB. Size histograms of (B) AuNPs@Cys and (E) AuNPs@CTAB. TEM images of (C) AuNPs@Cys and (F) AuNPs@CTAB after conjugating with *S. enteritidis.* (G) UV–vis absorbance spectra of AuNPs@Cys (black), *S. enteritidis* (red) and AuNPs@Cys-*S. enteritidis* (blue). (H) UV–vis absorbance spectra of AuNPs@CTAB (black), *S. enteritidis* (red) and AuNPs@CTAB-*S. enteritidis* (blue). The inset images: photo images of colorimetric change upon addition of *S. enteritidis*. (I) Zeta potentials of 1: AuNPs@Cys, 2: AuNPs@CTAB, 3: *S. enteritidis*, 4: *E. coli* O157, 5: AuNPs@Cys-*S. enteritidis*, 6: AuNPs@CTAB-*S. enteritidis*, 7: AuNPs@Cys-*E. coli* O157 and 8: AuNPs@CTAB-*E. coli* O157. (For interpretation of the references to colour in this figure legend, the reader is referred to the web version of this article.)

The nanoprobe is a key factor affecting the performance of this biosensor. Fig. 1A, B, D and E show the shape and size of the prepared AuNPs@Cys and AuNPs@CTAB, respectively. From TEM images of the prepared AuNPs, uniform and spherical shape was observed with average diameter of 26.4 nm (AuNPs@Cys) and 38.7 nm (AuNPs@CTAB). Fig. 1C and F present the TEM images after employing the AuNPs@Cys and AuNPs@CTAB nanoparticles to target bacteria (*S. enteritidis*) in an aqueous solution, which clearly showed that AuNPs probes were capable of binding to the surfaces and the lateral ends of *S. enteritidis* cells. Furthermore, suspensions containing bacteria showed that a larger number of AuNPs@Cys adhered to *S. enteritidis*, whereas a lower number of AuNPs@CTAB adhered to *S. enteritidis*. The phenomenon was attributed to the fact that AuNPs with different positive charges would have different strength of electrostatic interaction with the negatively charged bacteria. Fig. 1G and H show UV–vis spectra and images of probes and probes-bacteria complex. The dispersions of AuNPs@Cys changed from deep red to deep gray after the reaction with *S. enteritidis* (inset in Fig. 1G), and the dispersions of AuNPs@CTAB changed from light red to light purple after the reaction with *S. enteritidis* (inset in Fig. 1H), respectively. From Fig. 1G and H, it can be seen that bacteria with negatively charged surfaces such as *S. enteritidis* could cause color changes of (+) AuNPs, suggesting that surface charges played a vital role in bacteria identification (Li et al., 2017). The UV–vis spectrum of the AuNPs@Cys red-shifted from 522 to 531 nm and that of AuNPs@CTAB changed from 530 to 540 nm after the addition of bacteria, which might be caused by the adhesion interaction between AuNPs densely and the surfaces of the bacteria. Moreover, the zeta potential of AuNPs@Cys (in aqueous solution, pH = 7.0) was tested and exhibited strong positive potential, whereas the AuNPs@CTAB (in aqueous solution, pH = 7.0) exhibited a weak positive electric potential. Besides, bacteria displayed a negative potential, indicating that positively charged AuNPs can be adsorbed on the surface of bacteria through electrostatic interaction (Fig. 1I).

### 3.3 Optimization of assay conditions for the strip test

**Fig. 2:**
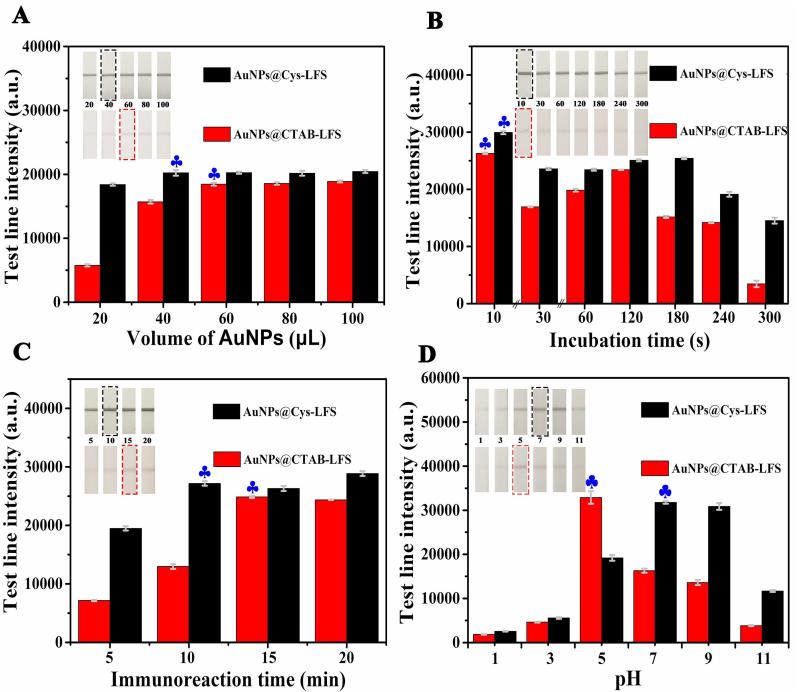
Effects of the (A) volume of AuNPs@Cys and AuNPs@CTAB, (B) incubation time, (C) immunoreation time and (D) different pH on LFS response for *S. enteritidis* of 10^8^ CFU/mL. Black and red rectangular boxes showed the optimal conditions of AuNPs@Cys-LFS and AuNPs@CTAB-LFS chosen for further experiments, respectively.

This novel biosensor was based on the color change of the AuNPs caused by the accumulation of *S. enteritidis*-AuNPs, resulting in a color enhancement of the *T*-line. Thus, the amount of AuNPs is an important factor to improve the performance of this optical biosensor, which was designed from 20 to 100 μL by incubation with *S. enteritidis*. In Fig. 2A, the results demonstrated that the *T*-line color of AuNPs@Cys-LFS deepened with AuNPs@Cys from 20 to 40 μL. A constant level of color brightness can be obtained when the volume of AuNPs@Cys reached to 40 μL. In addition, we observed that the *T*-line color of AuNPs@CTAB-LFS deepened with AuNPs@CTAB from 20 to 60 μL and kept almost consistent from 60 to 100 μL. Hence, the AuNPs@Cys (40 μL) and AuNPs@CTAB (60 μL) were set as the optimal candidates for LFS immunoassay.

Simultaneously, the incubation time of (+) AuNPs and *S. enteritidis* was also optimized. The mixture of AuNPs and microorganism was selected to react for 10, 30, 60, 120, 180, 240 and 300 s. As represented in Fig. 2B, the color bands of test zones weakened gradually with the increase of incubation time. Maybe the long reaction time between absorbent and bacteria could make the resultant conjugate sink, resulting in a hard chromatographic movement of the reaction solution. Thus, 10 s was taken as optimal incubation time, which was favorable to obtain the best performance of the (+) AuNPs-LFS.

Additionally, to obtain clearly visible *T*-lines, the immunoreaction time was also carefully optimized. As seen from Fig. 2C, the *T*-line signal increased obviously with the increasing immunoreaction time, and kept almost consistent at 10 min and 15 min respectively for AuNPs@Cys-LFS and AuNPs@CTAB-LFS, suggesting that the binding between McAb and *S. enteritidis* had tended to be saturated under that condition. Hence, 10 and 15 min were selected as the final immunoreaction time of AuNPs@Cys-LFS and AuNPs@CTAB-LFS, respectively.

The pH had significant influence on the sensitivity of immunosensor and the activity of the antibodies or antigens (Focke, Wnek, & Wei, 1987). To further verify the possibility of electrostatic interaction between (+) AuNPs and *S. enteritidis* at different pH conditions, zeta potentials of *S. enteritidis*, AuNPs@CTAB and AuNPs@Cys were carried out at pH 1, 3, 5, 7, 9 and 11. In Fig. S5, the zeta potentials of *S. enteritidis* were all negative charges, while it tended to positive charges for AuNPs@CTAB and AuNPs@Cys. These results proved that the bacteria and (+) AuNPs could be combined at any pH conditions. In addition, 10^8^ CFU/mL *S. enteritidis* solution of different pH (1, 3, 5, 7 and 9) was respectively mixed with a fixed concentration of AuNPs@Cys (40 μL) and AuNPs@CTAB (60 μL). As depicted in Fig. 2D, the highest color signal on *T*-line was obtained at pH 7 (AuNPs@Cys-LFS) and 5 (AuNPs@CTAB-LFS). Therefore, the best pH was chosen as 7 (AuNPs@Cys-LFS) and 5 (AuNPs@CTAB-LFS), respectively. More importantly, the test strip had better tolerance under extreme pH conditions than traditional gold-labeled antibody test strip (Fig. S6). In the case of pH was 1, 3 and 11, the proposed test strip could still detect the target, while the conventional test strip was no longer colored.

### 3.4 Sensitivity and selectivity of the biosensors

**Fig. 3:**
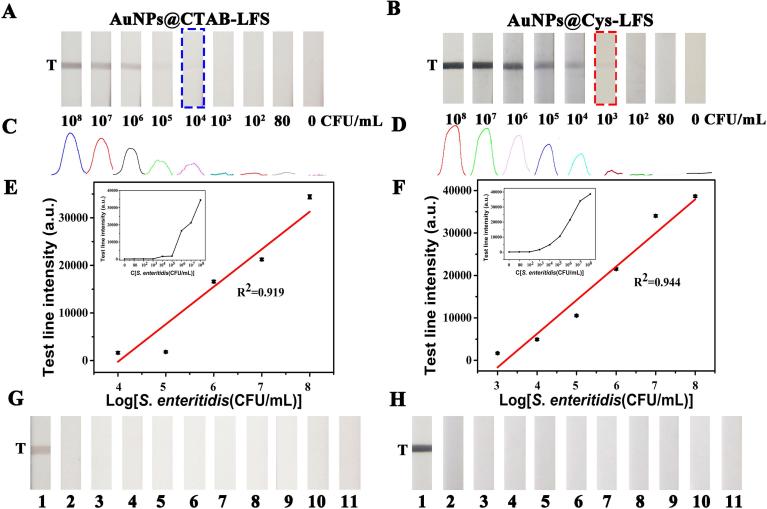
Assay performances of the AuNPs@CTAB-LFS (left) and AuNPs@Cys-LFS (right) biosensors for *S. enteritidis* detection. (A) and (B), results for sensitivity test. (C) and (D), quantitative results of the (+) AuNPs-based biosensor analyzed by Image J. (E) and (F), calibration curves of *S. enteritidis* detection separately using the AuNPs@CTAB-LFS and AuNPs@Cys-LFS (the inset image: the variation rule in the whole detection range). (G) and (H) Specificity research results from different interfering bacteria strains (Nos. 1–11 correspond to 10^8^ CFU/mL of *S. enteritidis*, *S. london*, *S. typhimurium*, *S. paratyphi B*, *S. hadar*, *S. aureus*, *E. coli*, *C. albicans*, *L. monocytogenes*, *C. coli* and control, respectively).

To evaluate the sensitivity of the biosensor, *S. enteritidis* standard aliquots (100 μL) with concentrations ranging from 0 to 10^8^ CFU/mL were analyzed by the AuNPs@CTAB and AuNPs@Cys based LFS under optimized conditions, respectively. The evaluation was conducted by the naked eyes and strip software that read intensity. Results showed that the changing of color on *T*-line continuously deepened with the *S. enteritidis* concentration increasing from 10^4^ to 10^8^ CFU/mL using AuNPs@CTAB (Fig. 3A), and the color of *T*-line deepened with increasing the concentration of *S. enteritidis* from 10^3^ to 10^8^ CFU/mL using AuNPs@Cys (Fig. 3B). In Fig. 3A and B, the images revealed that *S. enteritidis* concentrations at or above 10^4^ and 10^3^ CFU/mL were readily detectable using AuNPs@CTAB and AuNPs@Cys based LFS, respectively. The corresponding optical densities at the *T*-line were further recorded with Image J (Fig. 3C and D) (Yao et al., 2016). A linear relationship between *T*-line intensity of AuNPs@CTAB-LFS and concentration of *S. enteritidis* was obtained in the range between 10^4^ and 10^8^ CFU/mL, Y = 8634.85X–36321.26 (X = log [*S. enteritidis* concentration], R^2^ = 0.919); For AuNPs@Cys-LFS, a linear relationship was observed from 10^3^ to 10^8^ CFU/mL, as descripted in equation of Y = 7915.73X–25409.22 (R^2^ = 0.944). To further verify the excellent sensitivity of the proposed adsorption LFS system, as indicated from Table 1, compared with other materials-labeled recognition molecules test strips for *Salmonella* detection, the detection limit of the novel biosensor could surpass those of most reports.

**Table 1:** Comparison of sensitivity for *Salmonella* detection by LFS based on different signals.

Label	Target pathogen	LOD^a^ (CFU/mL)	Reference
GMBNs^b^-Ab^c^	*S. choleraesuis*	10^5^	Xia, Yu, Liu, Xu, and Lai (2016)
Au/MNCs^d^-Ab	*Salmonella*	10^3^	Hwang, Kwon, Lee, and Jeon (2016)
MNPs^e^-Ab	*S. enteritidis*	1.95 × 10^5^	Duan et al. (2017)
AuNPs-Ab	*S. typhimurium*	1.25 × 10^5^	Wang et al. (2015)
AuNPs-Ab	*S. typhimurium*	1.47 × 10^4^	Shin et al. (2018)
AuNPs-Ab	*S. typhimurium*	10^4^	Singh, Sharma, and Nara (2015)
AuNPs-Ab	*Salmonella*	10^3^	Wang et al. (2016)
HRP^f^-Ab	*S. typhimurium*	9.2 × 10^3^	Park, Yong, and Kim (2010)
CFMNs^g^-Ab	*S. typhimurium*	3.5 × 10^3^	Hu et al. (2018)
(+) AuNPs	*S. enteritidis*	10^3^	This work

a LOD, limit of detection.

b GMBNs, gold magnetic bifunctional nanobeads.

c Ab, antibody.

d Au/MNCs, gold-coated magnetic nanoparticle clusters.

e MNPs, magnetic nanoparticles.

f HRP, horseradish peroxidase.

g CFMNs, colorimetric-fluorescent-magnetic nanospheres.

Control experiments using traditional AuNPs-McAb based LFS was then conducted to compare the performance with the developed adsorption biosensor. The preparation process of traditional AuNPs and AuNPs-McAb, optimization and detection processes of traditional LFS were shown in the Supplementary materials. Serial concentrations of *S. enteritidis* (0–10^8^ CFU/mL) were analyzed using the same procedures as aforementioned. As shown in Fig. S7, the sensitivity was obtained at 10^6^ CFU/mL for the traditional AuNPs-McAb based LFS biosensor, showing that the proposed unlabeled probe possessed a stronger binding capacity for bacteria. So, at least 100 or 1000-fold improvement of sensitivity was achieved by using (+) AuNPs as the recognition agent instead of AuNPs-McAb.

To confirm the specificity of the biosensor, the 10^8^ CFU/mL *S. enteritidis* and other similar strains including nine strains as the interference pathogens were carried out. According to the results (Fig. 3G and H), the other bacteria exhibited consistent of no color compared with the control solution, only the *S. enteritidis* gave a bright color on *T*-line, indicating remarkable selectivity of two biosensors for *S. enteritidis*. The excellent selectivity maybe attributed to the specific McAb which was elicited by *Salmonella* flagellin in the animals (Li, Liu, Han, & Yan, 2012).

Furthermore, to confirm the universal property of the biosensor to other microorganism, *E. coli* O157 was employed as another model and detected using the developed (+) AuNPs-LFS (*T*-line was coated with anti-*E. coli* O157 McAb). In Figs. S8 and S9, it can be concluded that the proposed (+) AuNPs-LFS could also be used for detecting *E. coli* O157 with a high sensitivity (10^5^ CFU/mL of AuNPs@Cys-LFS and 10^4^ CFU/mL of AuNPs@CTAB-LFS) and selectivity.

### 3.5 Application in water, lettuce and pork samples

**Fig. 4:**
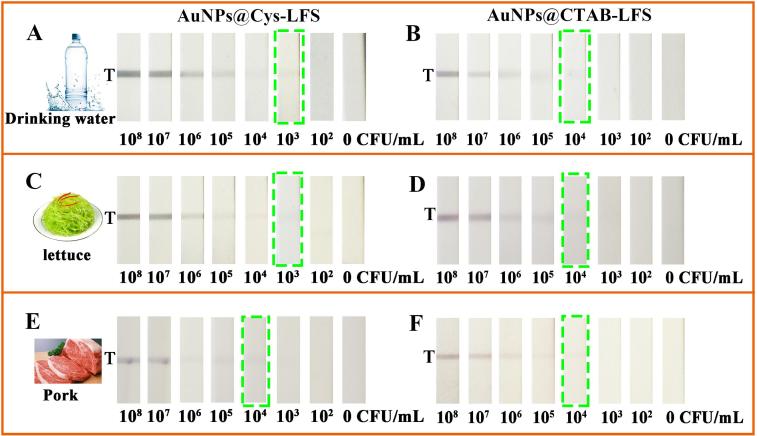
Detection results of *S. enteritidis* in water, lettuce and milk samples separately by the AuNPs@Cys-LFS (A, C, E) and AuNPs@CTAB-LFS (B, D, F) biosensors.

The practical applicability of the device was tested by detecting the spiked *S. enteritidis* and *E. coli* O157 in drinking water, lettuce and pork samples at a final concentration of 10^2^, 10^3^, 10^4^, 10^5^, 10^6^, 10^7^ and 10^8^ CFU/mL and non-spiked samples. As seen from Fig. 4A–D, for drinking water and lettuce samples, sensitivity were all obtained at 10^3^ CFU/mL (AuNPs@Cys-LFS) and 10^4^ CFU/mL (AuNPs@CTAB-LFS), respectively. For pork samples, the color signal on the T zone all could be observed at 10^4^ CFU/mL for two (+) AuNPs-LFSs (Fig. 4E and F). Therefore, the sensitivities of the proposed immunosensor for these three samples were almost consistent with values determined in section 3.4, showing that the biosensor was valid for *S. enteritidis* with little matrix effects on the results. Moreover, the two novel biosensors were also used to analyze *E. coli* O157 in food samples. In Fig. S10, the sensitivity for *E. coli* O157 were all 10^4^ CFU/mL and 10^5^ CFU/mL separately for AuNPs@Cys-LFS and AuNPs@CTAB-LFS in drinking water, lettuce and pork samples. In addition, the zeta potentials of the bacteria, (+) AuNPs and (+) AuNPs-bacteria in these samples were measured. In Fig. S11, it was found that their values decreased slightly due to the influence of the matrix, but (+) AuNPs were still positive charged, and the bacteria were negatively charged, indicating that the bacteria and (+) AuNPs could be combined in actual samples. Thus, the outstanding performance demonstrated that the robust LFS biosensor holds great promise as reliable tool for the detection of foodborne bacteria in real samples.

## 4 Conclusions

We have constructed a lateral flow immunoassay based on diverse surface charged AuNPs (AuNPs@Cys and AuNPs@CTAB), which could give a rapid and differential response to bacteria. The approach exploits the ability to change the adsorption efficiency by adjusting the surface electronic properties of AuNPs. Adoption of label-free AuNPs and antibody as recognition agent carriers on *T*-line allowed highly sensitive and selective detection of *S. enteritidis*. The detection range of the developed assay can be as wide as 10^3^–10^8^ cells, and the detection limit can be as low as 10^3^ CFU/mL. Most importantly, the developed (+) AuNPs-LFS could also be used for screening another model target (*E. coli* O157), possessed a universal applicability for detection of foodborne pathogens. Our study revealed that diversely (+) AuNPs are attractive as versatile probes for various applications in the fields of clinical diagnostics and food contamination monitoring.

## Declaration of Competing Interest

The authors declare that they have no known competing financial interests or personal relationships that could have appeared to influence the work reported in this paper.
