# Supplementary material for: Diversely positive-charged gold nanoparticles based biosensor: A label-free and sensitive tool for foodborne pathogen detection
Source: Food Chem X. 2019 Aug 23;3:100052. doi: 10.1016/j.fochx.2019.100052 (PMC6831733; doi:10.1016/j.fochx.2019.100052)
Supplement: Supplementary data 1 [file mmc1.doc]

**Supporting Information**

**Diversely positive-charged gold nanoparticles based biosensor: A label-free and sensitive tool for foodborne pathogen detection**

Tong Bu^1,2^, Pei Jia^1,2^, Jinghan Liu^2^, Xinyu Sun^2^, Meng Zhang^2^, Yongming Tian^2^, Daohong Zhang^2^, Jianlong Wang^2^, Li Wang^2*^, Qingyou Lu^1,3*^, Jihao Wang^1*^

1. Key Laboratory of Condensed Matter Physics at Extreme Conditions, High Magnetic Field Laboratory of the Chinese Academy of Sciences, Hefei 230031, China

2. College of Food Science and Engineering, Northwest A&F University, Yangling 712100, Shaanxi, China

3. Hefei National Laboratory for Physical Sciences at the Microscale, University of Science and Technology of China, Hefei 230026, China

* Author for correspondence:

Tel.: +86 29-8709-2486

Fax: +86 29-8709-2486

lwang@nwsuaf.edu.cn, qxl@hmfl.ac.cn and jihaowang@hmfl.ac.cn

*Preparation of traditional colloidal gold*

In this assay, colloidal gold solution was prepared by using trisodium citrate to reduce HAuCl_4_·3H_2_O according to the classical Frens . One milliliter of 1% hydrogen tetrachloroaurate trihydrate was added to 96 mL of ultrapure water, stirred, and heated to boiling temperature. At this time the color was yellowish green. Four milliliters of 1% trisodium citrate was added to the mixture immediately and the color changed from yellowish green to colorless. The reaction was maintained about 10 min at boiling temperature until the color changed to stable and transparent red, then stirred for another 15 min. Finally, the solution was cooled at room temperature and stored at 4 ℃ for further use.

*Synthesis of the AuNPs-McAb conjugate*

AuNPs-McAb was prepared according to the method reported previously . The pH of the colloidal gold solution was adjusted by the addition of 0.01 M K_2_CO_3_. Under gentle stirring, anti-*S. enteritidis* McAb was added to the colloidal gold solution, agitated for 30 min, then 10% BSA solution was mixed with the colloidal gold solution, and used a final concentration of 1% BSA to block the residual sites on the surface of AuNPs. The mixed solution was kept stirring for 30 min and stored for 2 h at 4 ℃. Then, in order to remove unconjugated McAb and BSA, this solution was centrifuged at 208 ×g for 15 min. The pellets of AuNPs were collected by centrifuging at 13,363 ×g for 35 min and the soft sediment was washed one time with 2 mM borate buffer. Finally, the obtained AuNPs were redispersed in borate buffer and stored at 4 ℃ for further using.

*Preparation of the traditional AuNPs-McAb based LFS*

Preparation of the traditional AuNPs-McAb based LFS were identical to the experimental procedures described in Section 2.5. The difference was that the nanogold probe particles were dispensed onto the conjugate pad at a proper jetting rate and dried with a vacuum freeze drier for 3 h.

*Detection process of the traditional AuNPs-McAb based LFS*

The inactivated *S. enteritidis* bacterial solution was serially diluted to 0–10^8^ CFU/mL in 10 mM PBS (pH 7.4), with PBS as the control solution. 100 μL of *S. enteritidis* solutions of different concentrations were added to the sample pad. The signal intensity on the test line was observed after 10 min. The minimum detectable concentration was defined as the sensitivity of the LFS. All experiments were performed in triplicates.

Bu, T., Huang, Q., Yan, L., Huang, L., Zhang, M., Yang, Q., Yang, B., Wang, J., & Zhang, D. (2018). Ultra technically-simple and sensitive detection fo*r Salmonella Enteritid*is by immunochromatographic assay based on gold growth*. Food Control,* 84, 536-543.

**Table S1.** Information for bacterial strains employed in this work

| Bacteria | Abbreviation | ATCC No. |
| --- | --- | --- |
| *Salmonella* *enteritidis Escherichia coli* O157  *Salmonella paratyphi B*  *Salmonella typhimurium*  *Salmonella hadar*  *Salmonella london*  *Staphylococcus aureus*  *Candida albicans*  *Listeria monocytogenes*  *Campylobacter coli* | *S. enteritidis*  *E. coli* O157  *S. paratyphi B*  *S.* *typhimurium*  *S. hadar*  *S. london*  *S. aureus*  *C. albicans*  *L. monocytogenes*  *C. coli* | 13076  43889  10719  14028  51956  8389  25923  96268  19114  43461 |

Characterization of McAb

The sensitivity and specificity of the McAb used in this work were characterized by an in-ELISA. According to the criterion , P/N ≥ 2.1 was positive and P/N < 2.1 was negative results (P represents the values of positive sample minus the blank, and N represents the values of negative sample minus blank). The sensitivities of McAbs for *S. enteritidis* and *E. coli* O157 detections were 10^3^ (black line) and 10^6^ (red line) CFU/mL, respectively. The analysis chart was shown in Fig. S1. Moreover, ELISA results (Fig. S2 and S3) showed that except for *S. enteritidis*, the P/N for other bacteria were ranged from 0.52 to 0.9 and except for *E. coli* O157, the P/N for other bacteria were ranged from 0.13 to 0.57 (data not shown) which were all below 2.1. In summary, the prepared antibodies used in this work performed high sensitivity and specificity.


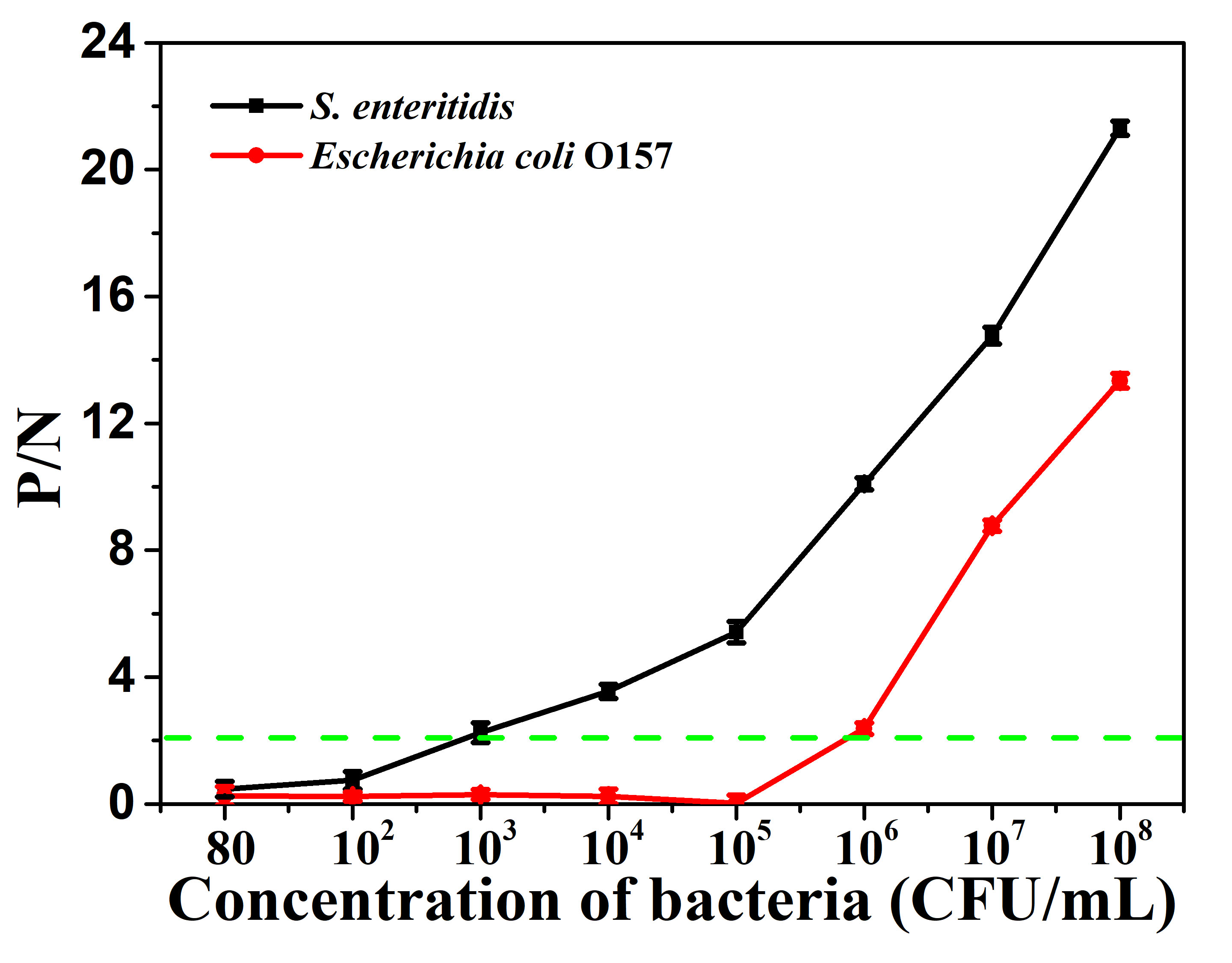


**Fig. S1** Sensitivity analysis curves of the McAbs for *Salmonella* and *E. coli* O157 detection by ELISA, separately. The green dash line indicates the criterion for result judgement of positive and negative.


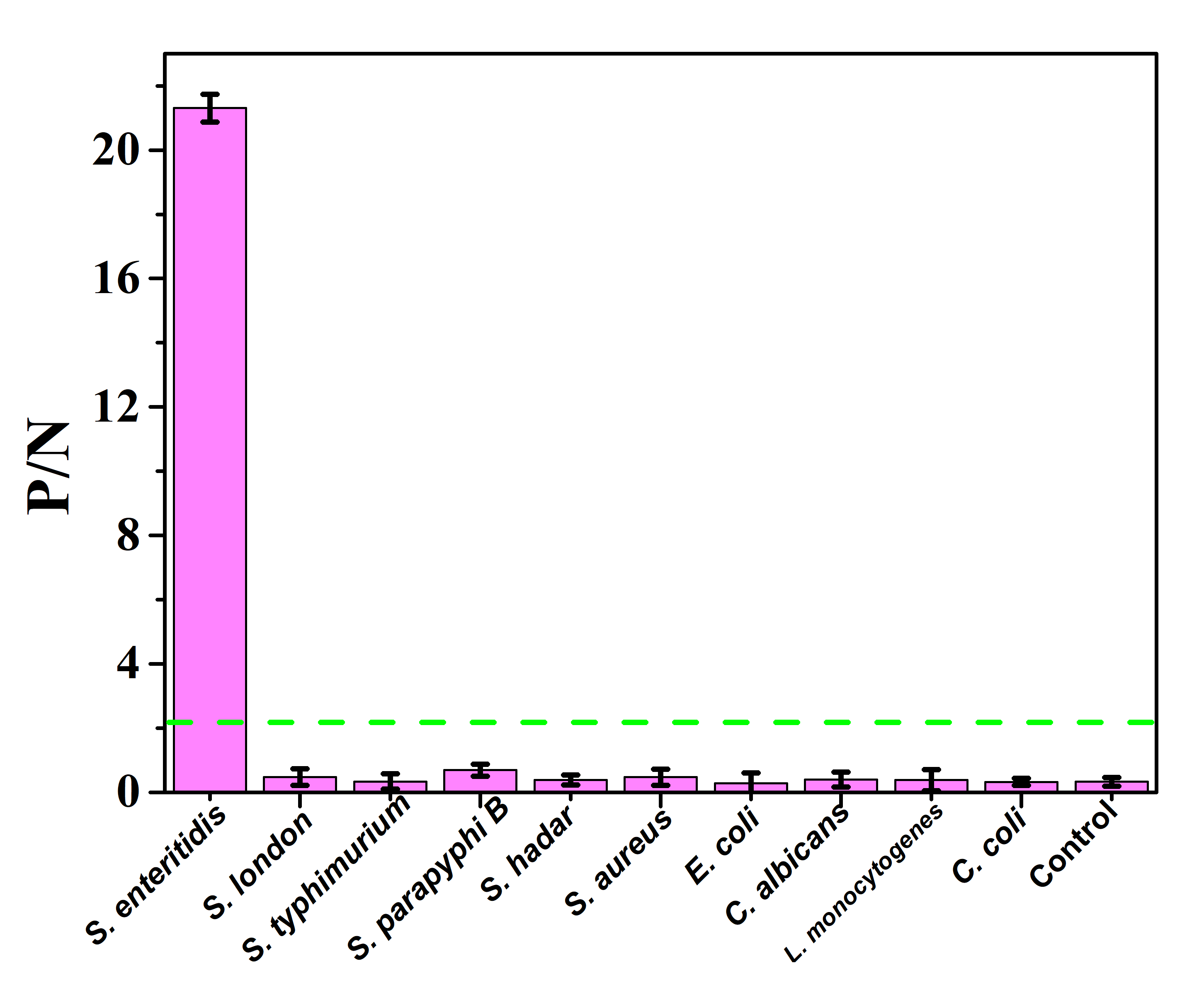


**Fig. S2** Specificity test results for pathogen bacteria detection using McAbs of *S. enteritidis*. The green dash line indicates the criterion for result judgement of positive and negative.


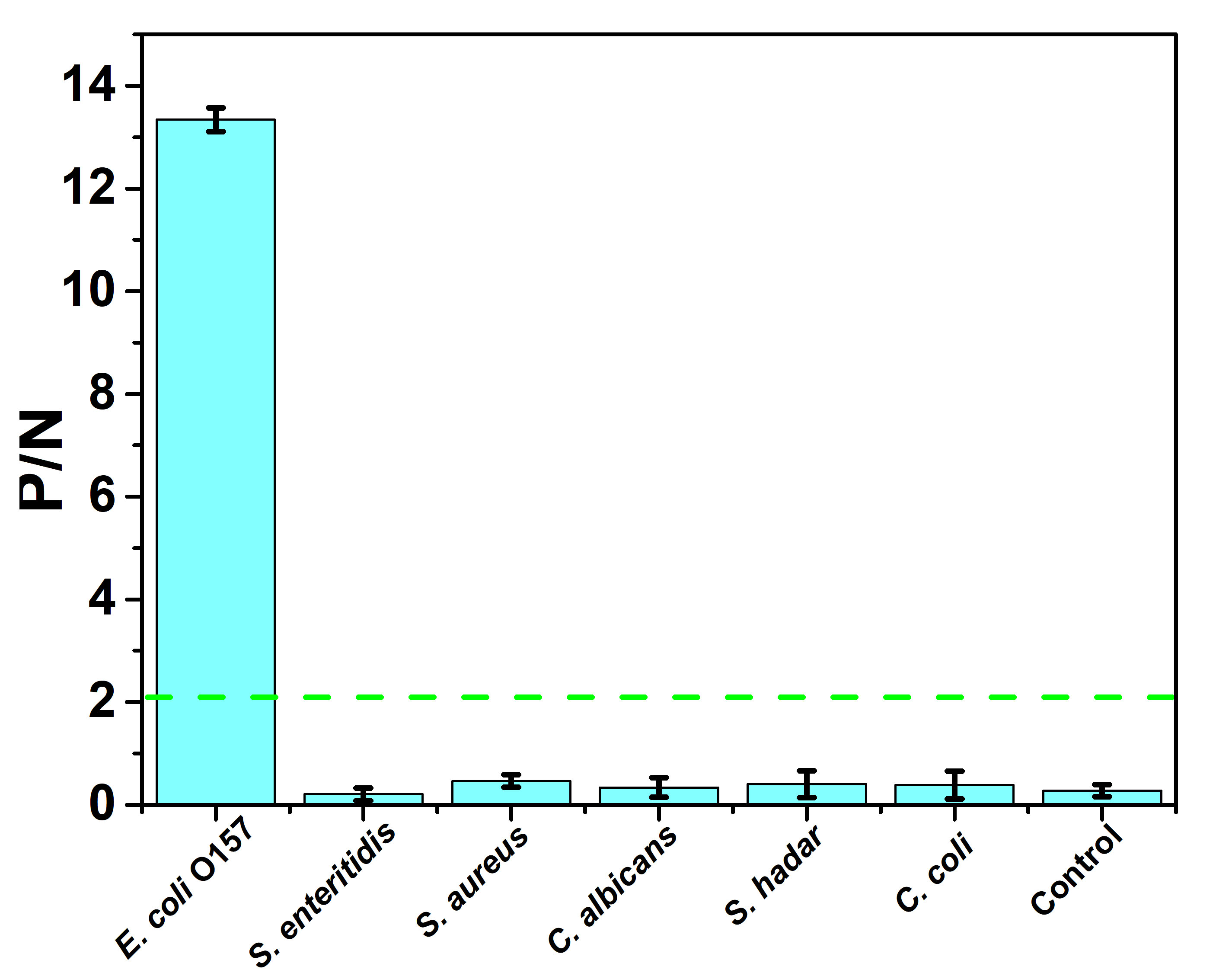


**Fig. S3** Specificity test results for pathogen bacteria detection using McAbs of *E. coli* O157. The green dash line indicates the criterion for result judgement of positive and negative.

Li, N. L., Feng, J. J., Jing, C., Fu, G. Y., & Zhong, Q. W. (2013). Detection of circulating antigen in serum of mice infected with Trichinella spiralis by an IgY–IgM mAb sandwich ELISA. *Experimental Parasitology, 133*(2), 150-155.


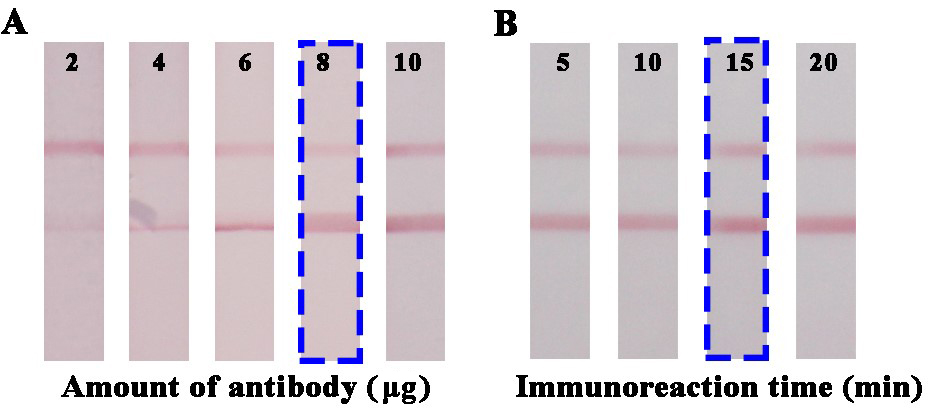


**Fig. S4** Effects of (A) amount of antibody and (B) immunoreaction time on traditional AuNPs-LFS responses for *S. enteritidis* of 10^8^ CFU/mL. Blue rectangular boxes: the optimal conditions chosen for further experiments.

*Optimization of assay conditions for the traditional strip test.*

The amount of antibody played an important role in the detection performance of the strip platform. In detail, 2, 4, 6, 8 and 10 μg of antibody were labeled on traditional AuNPs. The optimization results were shown in Fig. S4A, with the increase of antibody quantity, the color intensity on T-line enhanced gradually until the antibody raised to 8 μg, and thereafter kept at an almost constant level. Thereby, 8 μg was considered to be the optimal amount of antibody. Simultaneously, influence of immunoreaction time on the color development on T-line of the strip was also examined. The test strip was allowed to react for 5, 10, 15 and 20 min. As shown in Fig. S4B, with the increase of reaction time, the color intensity on T-line enhanced until the time reached 15 min, and then reached a plateau. Thereby, 15 min was considered to be the optimal immunoreaction time.


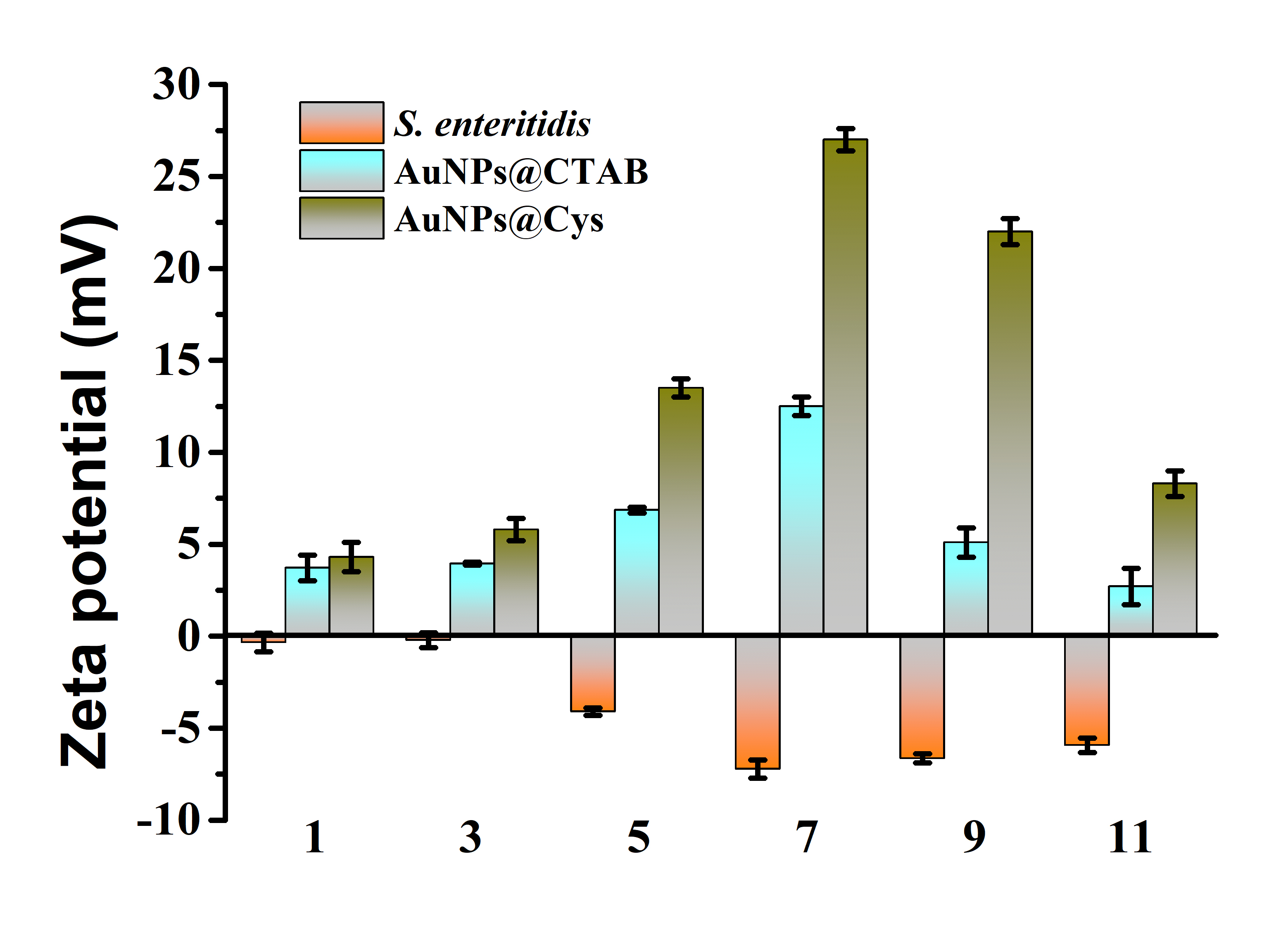


**Fig. S5** Zeta Potentials of *S*. *enteritidis*, AuNPs@CTAB and AuNPs@Cys at different pH conditions.


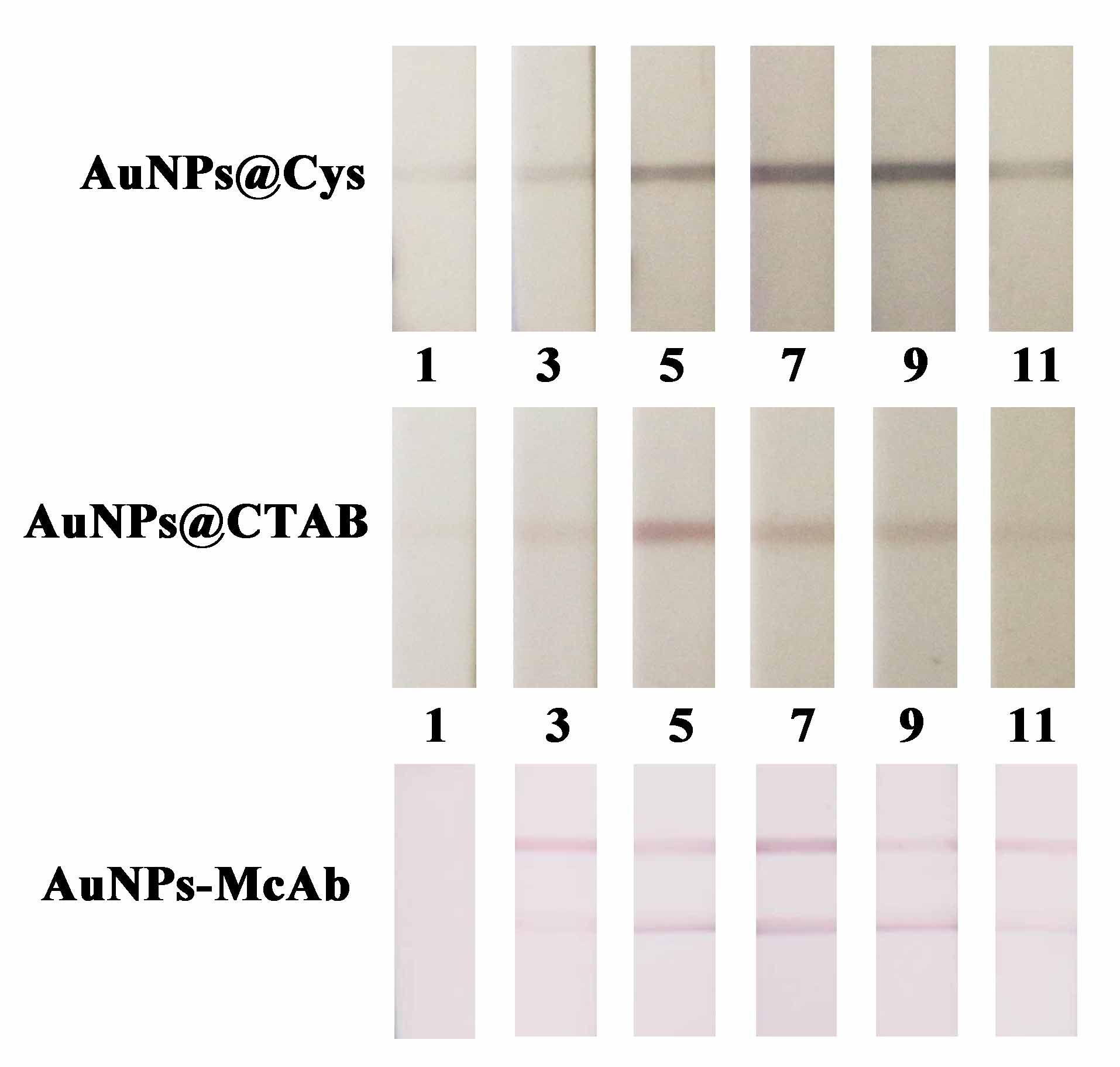


**Fig. S6** AuNPs@Cys, AuNPs@CTAB and traditional AuNPs-McAb based LFS for detection of *S. enteritidis* at different pH conditions, respectively.


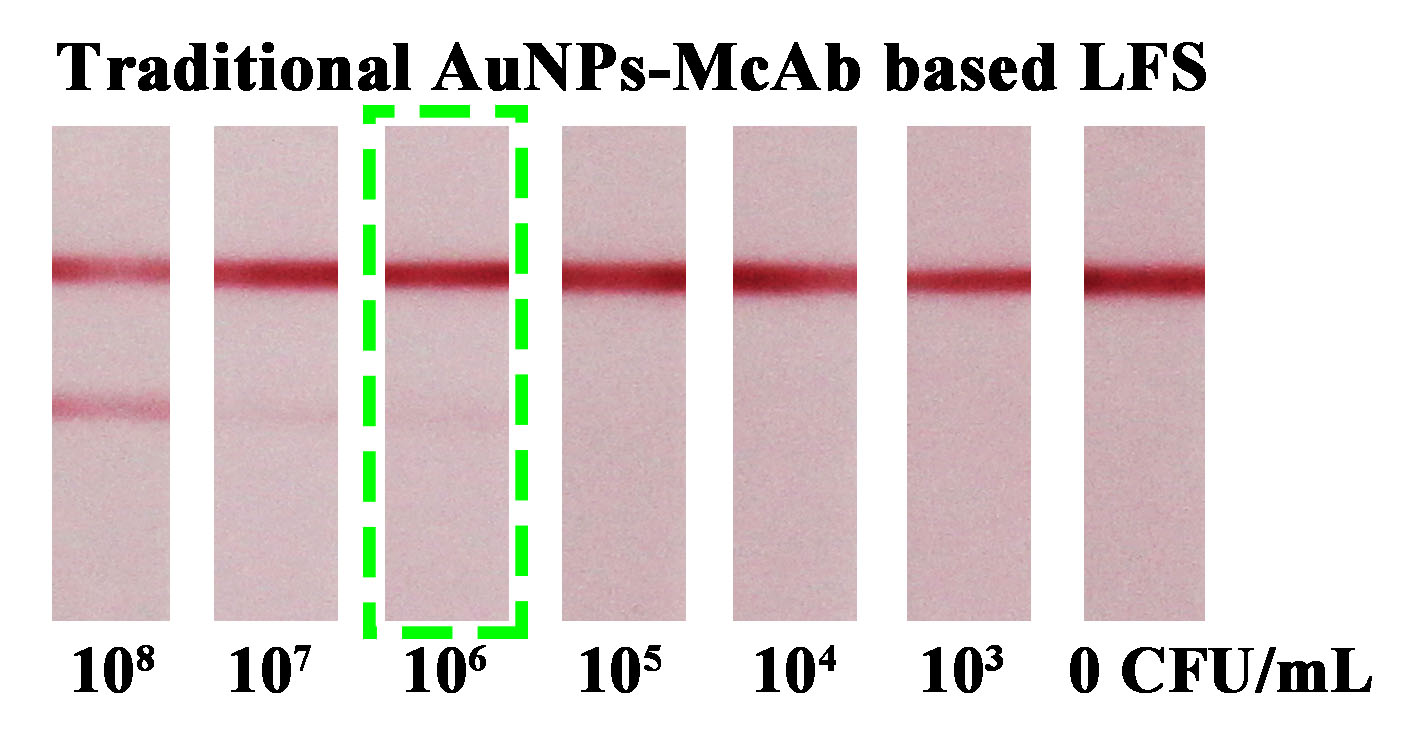


**Fig. S7** Results for sensitivity test of traditional AuNPs-McAb based LFS for *S*. *enteritidis* detection.


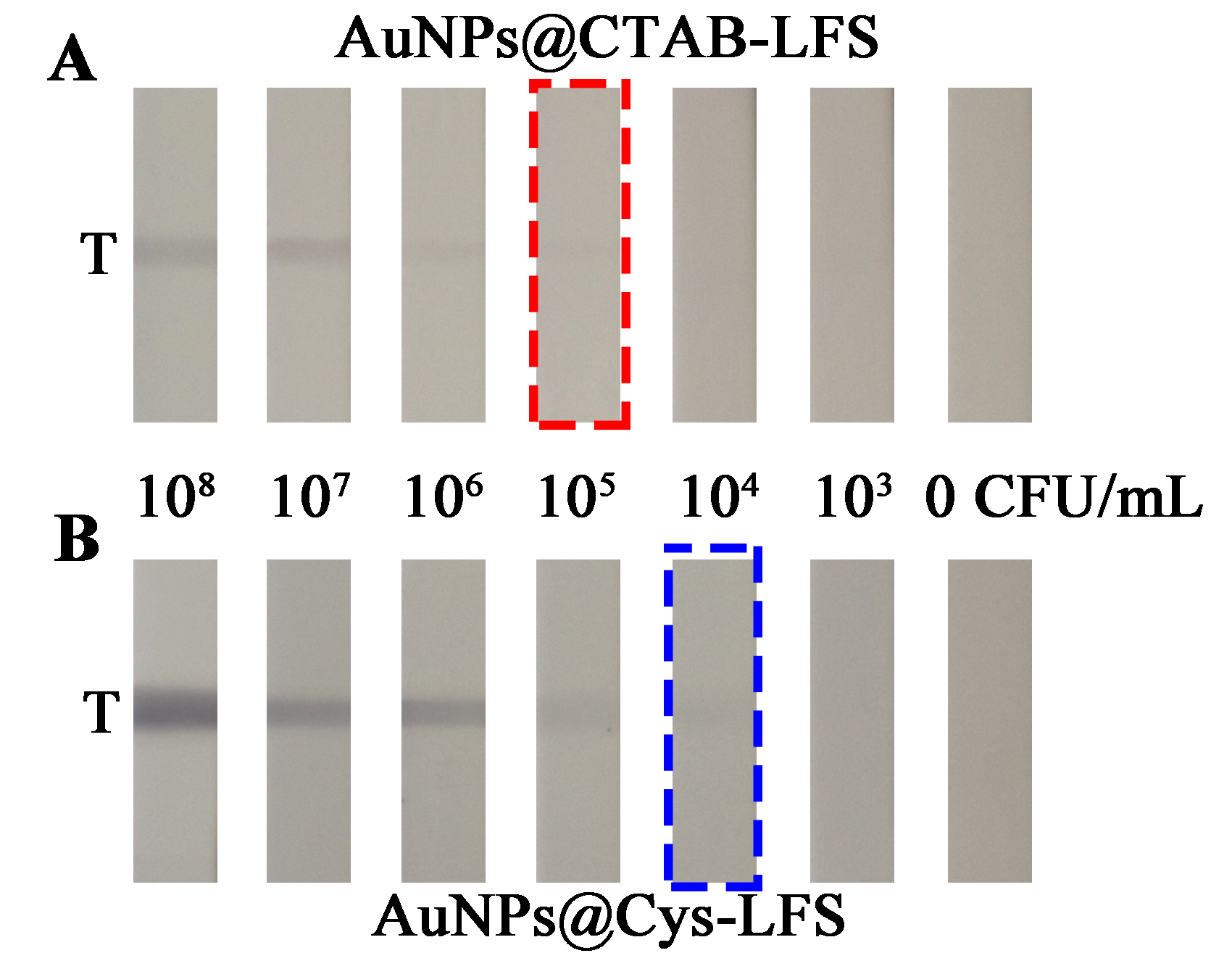


**Fig. S8** Results for sensitivity test of (A) AuNPs@CTAB-LFS and (B) AuNPs@Cys -LFS for *E.coli* O157 detection.


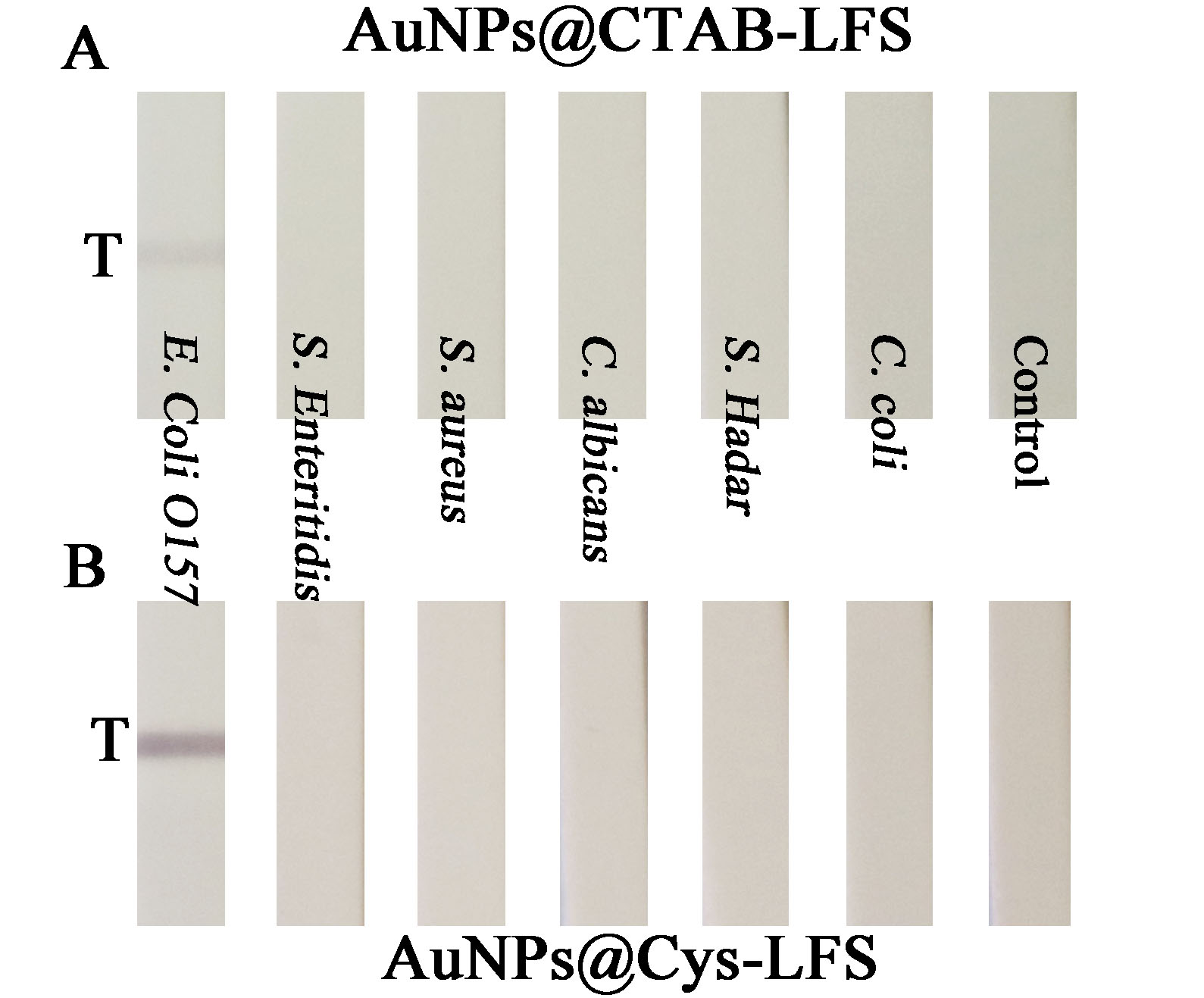


**Fig. S9** Results for specificity test of (A) AuNPs@CTAB-LFS and (B) AuNPs@Cys -LFS for *E.coli* O157 detection.


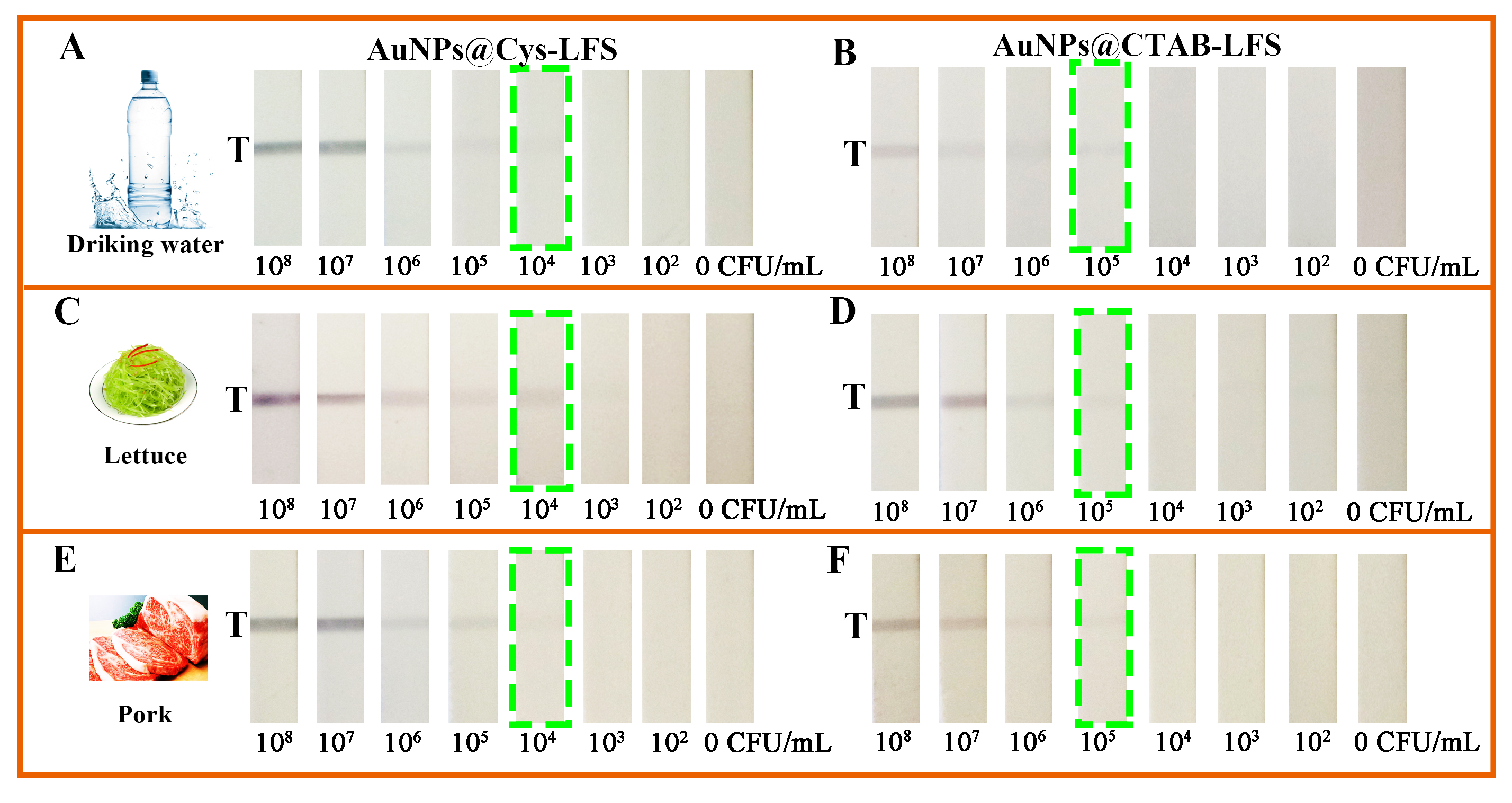


**Fig. S10** Detection results of *E. coli* O157 in water, lettuce and milk samples separately by the AuNPs@Cys-LFS (A, C, E) and AuNPs@CTAB-LFS (B, D, F) biosensors.


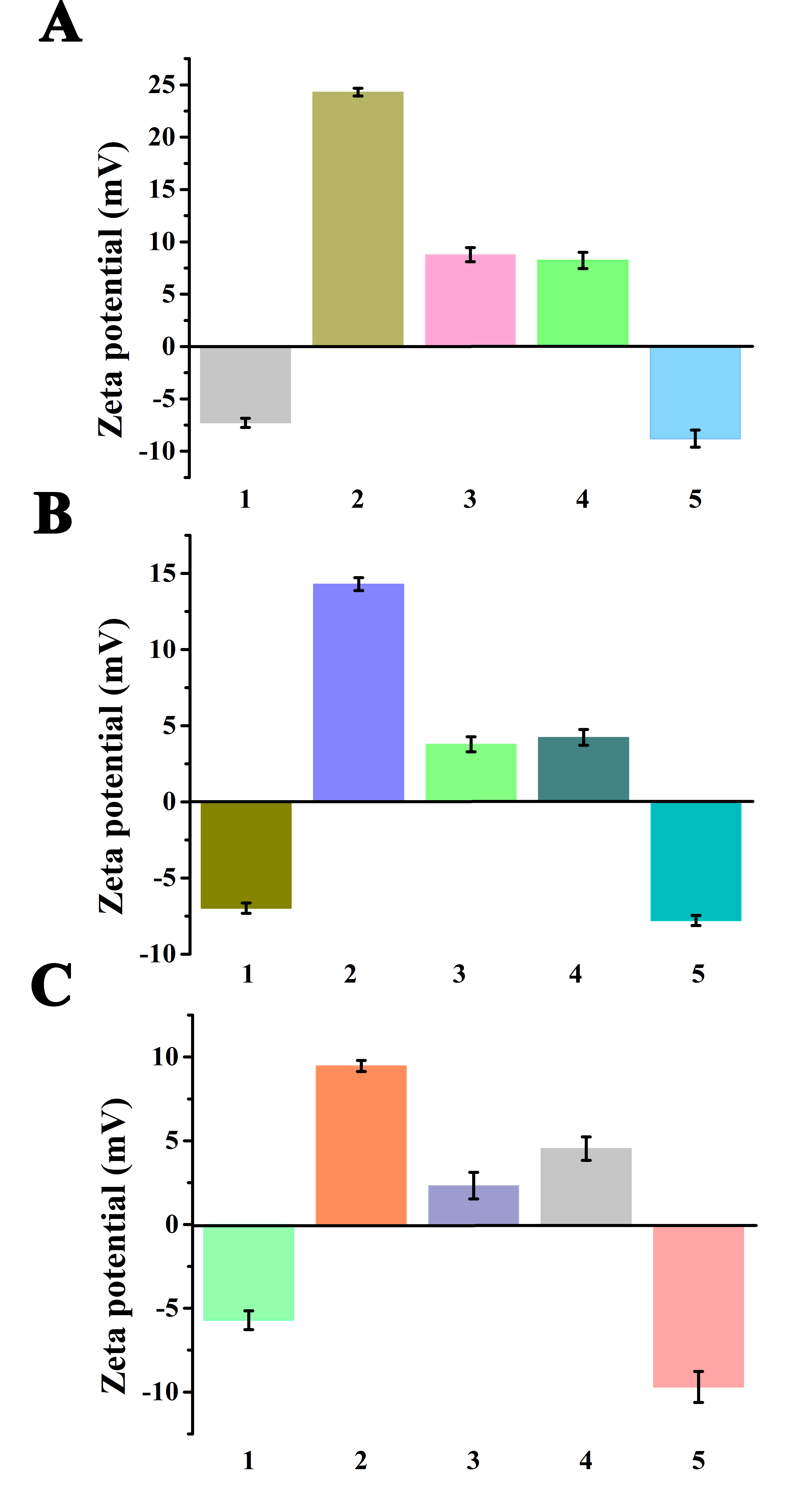


**Fig. S11** Zeta potentials of 1: *S. enteritidis*, 2: AuNPs@Cys, 3: AuNPs@CTAB, 4: AuNPs@Cys-*S. enteritidis* and 5: AuNPs@CTAB-*S. enteritidis* separately in (A) drinking water, (B) lettuce and (C) pork samples.
